# Cost‐Effectiveness of Venom Immunotherapy in Preventing Severe Bee and Wasp Sting Reactions

**DOI:** 10.1111/all.70176

**Published:** 2025-12-18

**Authors:** Gunter Sturm, Maria Beatrice Bilò, Carmen Vidal, Joanna N.G. Oude Elberink, Jochen Schmitt, Andreas Kallsoy Slættanes, Thilo Jakob

**Affiliations:** ^1^ Department of Dermatology and Venereology Medical University of Graz Graz Austria; ^2^ Allergy Outpatient Clinic Reumannplatz Vienna Austria; ^3^ Department of Clinical and Molecular Sciences Università Politecnica Delle Marche Ancona Italy; ^4^ Allergy Unit, Department of Internal Medicine University Hospital Ospedali Riuniti di Ancona Ancona Italy; ^5^ Allergy Department Complejo Hospitalario Universitario de Santiago, Faculty of Medicine USC Santiago de Compostela Spain; ^6^ Department of Allergology University Medical Center Groningen, University of Groningen, and Groningen Research Institute for Asthma and COPD Groningen the Netherlands; ^7^ Center for Evidence‐Based Healthcare TU Dresden Dresden Germany; ^8^ ALK‐Abelló A/S Hørsholm Denmark; ^9^ Department of Dermatology and Allergy University Medical Center, Justus‐Liebig University Gießen Germany

**Keywords:** immunotherapy vaccines and mechanisms, quality of life, venom and insect allergy

## Abstract

**Background:**

Systemic sting reactions (SSRs) from bee and wasp stings can cause severe symptoms, including anaphylaxis and potentially lead to fatal outcomes. These reactions can significantly affect individuals' daily lives due to the fear and anxiety associated with the risk of stings. Venom immunotherapy (VIT) has been shown to be an effective preventive treatment for SSRs, offering a viable alternative to emergency treatments like adrenaline auto‐injectors (AAIs).

**Methods:**

We created a decision tree framework with a Danish payer perspective designed to evaluate the cost‐effectiveness of VIT with Alutard^®^ SQ in individuals with bee and/or wasp venom allergies. Input in the model was identified from a structured literature review and expert consensus. The incremental cost‐effectiveness ratio (ICER) of Hymenoptera venom depot immunotherapy (HVDI) compared to AAIs over a 10‐year time horizon was estimated. The impact of uncertainty associated with key assumptions was investigated using univariate deterministic sensitivity analyses (DSA).

**Results:**

In the base case, the incremental cost of HVDI was €7428, while the incremental quality‐adjusted life years (QALYs) gained were 0.48. Consequently, the ICER (cost per QALY) of HVDI compared to AAIs after 10 years was estimated at €15,550.

**Conclusion:**

VIT is a cost‐effective treatment for the prevention of severe reactions to wasp and/or bee stings when compared to AAIs.

## Graphical abstract

This study evaluates the cost‐effectiveness of Hymenoptera venom depot immunotherapy (HVDI) with Alutard SQ compared to adrenaline auto‐injectors (AAIs) in Denmark. The model incorporates both psychological and physiological benefits to estimate quality‐adjusted life years (QALYs). Venom immunotherapy (VIT) is a cost‐effective treatment for the prevention of severe reactions to wasp and/or bee stings when compared to AAIs. AAI, adrenaline auto‐injector; HVDI, Hymenoptera venom depot immunotherapy; ICER, incremental cost‐effectiveness ratio; QALY, quality‐adjusted life year; SSR, systemic sting reaction; VIT, venom immunotherapy.

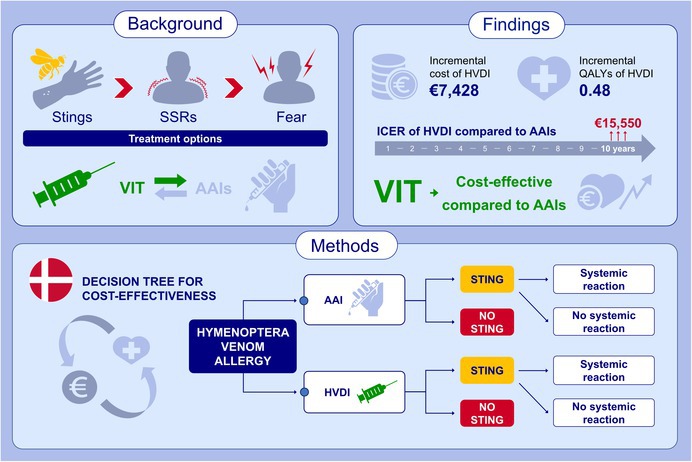

## 1 Introduction

Stings from insects with venom sacs, such as bees and wasps of the order Hymenoptera, most commonly cause localized burning, pain, erythema, and minor edema at the sting site. However, some individuals experience an allergic reaction to bee and/or wasp stings potentially causing SSRs with severe symptoms manifesting in anaphylaxis, impacting the respiratory or cardiac system, potentially culminating in a lethal outcome [1, 2, 3, 4, 5, 6]. SSRs are mediated by immunoglobulin E (IgE) directed against allergens of bee or wasp venom [7]. Hence, an allergic reaction can be a frightening experience, and consequently, individuals may limit their exposure to activities and jobs they consider associated with the risk of a sting, causing restrictions on their daily life [6, 8, 9]. The prevalence of sensitization to Hymenoptera venom in adults in the general population is estimated to vary from 9.3% up to 41.6% [10, 11]. The prevalence of individuals experiencing SSRs in Europe has been reported to range between 0.3% and 7.5% [12]. The European Academy of Allergy and Clinical Immunology's (EAACI) guideline on Hymenoptera venom allergy describes two different treatment strategies: emergency medication (e.g., AAIs) administered immediately after a SSR occurs and VIT, as a treatment to prevent further SSRs [6, 13].

Current EAACI guidelines state that VIT should be continued for at least 3–5 years depending on severity, while Danish guidelines recommend 5 years of treatment [6, 14]. HVDI is indicated for patients with bee or wasp venom allergy with previous generalized and/or systemic IgE‐mediated allergic reactions as a result of sensitization to bee or wasp venom, confirmed by skin test (prick test and/or intradermal test) and/or specific IgE test [15, 16, 17, 18].

The literature regarding health economic evaluation for VIT is scarce. However, Hockenhull et al. [4] conducted a systematic review to investigate the cost of VIT using (now discontinued) Pharmalgen as a case. The study estimated the costs and resource utilization associated with VIT in the United Kingdom (UK). Hence, to our knowledge, our analysis of the cost‐effectiveness of VIT from a Danish perspective is the first evaluation to include the combined psychological and physiological benefit of VIT.

## 2 Methods

### 2.1 Structured Literature Review

A structured literature review was conducted to identify relevant data for the cost‐effectiveness model. Two population, intervention, comparator, outcome (PICO) scenarios were defined to identify information related to the costs and resource use related to bee and/or wasp venom allergy. Table 1 presents an overview of the PICOs that have been utilized. The intervention and comparator were intentionally excluded to prevent an overly restrictive search that might omit pertinent data.

**TABLE 1:** “Cost search” and “resource use search.”

	Description—Cost search
Population	Individuals of all ages, including adults (aged 18 and over) and children (aged under 18), with bee and/or wasp sting allergies
Outcome	Cost‐effectiveness, cost of treatment, budgetary impact, immunization cost analysis, cost analysis, resource cost, direct costs, indirect costs, field sting, sting challenge

	Description—Resource use search
Population	Individuals of all ages, including adults (aged 18 and over) and children (aged under 18), with bee and/or wasp sting allergies
Outcome	Resource use, healthcare resource utilization, disease management, hospital resource, immunization resource, rush, ultra‐rush

Inclusion and exclusion criteria were defined as follows:

- Must be based on human subjects.
- Must be written in English.
- Must be from 1970 to 2023.

The searches were conducted across two databases, namely Embase and PubMed, on January 3, 2024 and on December 12, 2023. The full search strategies can be viewed in Appendices S1 and S2. The total amount of hits was first screened for duplicates, then by title, afterwards by abstract, and lastly by full text. The screening processes are outlined in Appendices S3 and S4. Based on the screening process, each search revealed two studies with potentially relevant information. The studies are presented in Appendices S5 and S6. Due to the low amount of identified studies, additional unstructured searches were conducted to gather data for the model where possible.

### 2.2 Model Framework

The economic model employed in this analysis is a decision tree framework with a Danish payer perspective designed to evaluate the cost‐effectiveness of VIT in individuals with bee and/or wasp venom allergies. The model compares HVDI to the use of AAIs by simulating the clinical pathways and associated costs over a 10‐year time horizon. Utilities are captured as decrements associated with fear. The structure of the model begins with an initial treatment decision node where patients can either receive VIT or rely solely on AAIs. The patients in the VIT arm receive an AAI during the up‐dosing phase. Subsequently, the model includes chance nodes representing the annual probability of being stung by a bee or wasp. If a sting occurs, additional chance nodes determine whether the patient experiences a nonsystemic reaction, which does not generate additional costs, or a systemic reaction, which generates additional costs linked to the treatment of the systemic reaction. The model's pathways lead to terminal nodes that encapsulate the final outcomes, including health states and associated costs.

**FIGURE 1:**
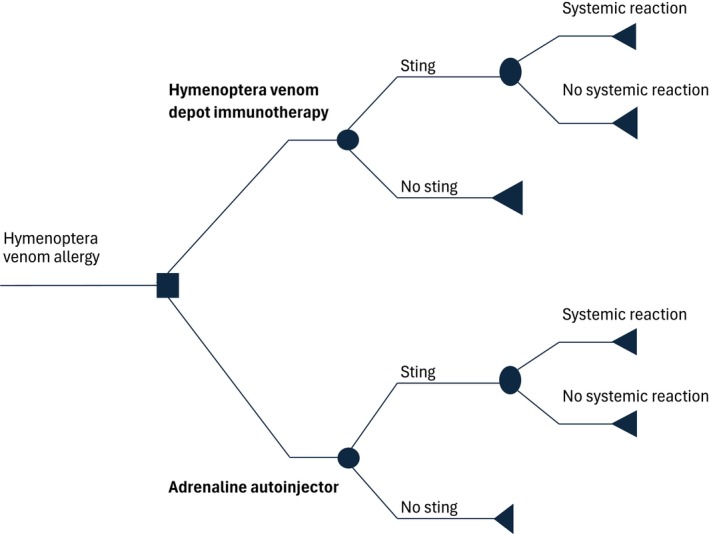
Structure of the health economic model.

The structure depicted in Figure 1 represents one cycle, corresponding to a single year in the model. For each year within the time horizon, the model applies the same structure but with different inputs. Specifically, year 1 includes the up‐dosing phase of VIT and a shorter maintenance phase, while subsequent years (year 1+) consist solely of the maintenance phase.

### 2.3 Model Inputs

Model inputs can be grouped into three key categories: efficacy of HVDI (based on Alutard SQ studies), utility associated with different events in the model, and cost associated with treatments.

Hockenhull et al. [4] estimated that the annual number of stings per person during or after treatment with VIT is 0.095. This input is used both in VIT and the AAI arm to estimate the annual risk of a sting. A 45% incidence rate of systemic reactions was chosen as an input in the model to estimate the likelihood of a systemic reaction in individuals allergic to bee and/or wasp stings based on a population susceptible to systemic reactions from Hymenoptera stings [19].

Table 2 provides an overview of the features and inputs in the health economic model.

**TABLE 2:** Features and inputs in the health economic model.

Feature	Input	Reasoning	Source
General			
Intervention	HVDI	Investigating the cost‐effectiveness of HVDI. Patients undergoing VIT, will receive an AAI during the up‐dosing phase, in alignment with Danish guideline	[14]
Comparator	Adrenaline auto‐injector: EpiPen	According to EAACI and Danish guidelines, patients with Hymenoptera venom allergy are advised to carry an adrenaline auto‐injector	[6, 14]
Country	Denmark	Setting based on the scope of the analysis	
Time horizon	10 years	In alignment with Danish guidelines, VIT should be administered for a duration of 5 years. A conservative estimate of the efficacy of VIT after treatment has been selected, assuming it will remain effective for 5 years. Consequently, a time horizon of 10 years has been selected, representing a conservative approach	[14]
Number of simultaneous prescribed AAI annually	1	To ensure a conservative perspective, it is assumed patients are prescribed 1 AAI at a time	
Up‐dosing phase—number of injections	15	Conventional up‐dosing phase, aligned with Summary of Product Characteristics (SmPCs). The number of injections corresponds to the number of weeks up‐dosing takes	[6, 16, 18]
Maintenance phase—injection interval (weeks)	6	Assumption of the mean amount weeks between injections during the maintenance phase, aligned with SmPCs	[6, 15, 17, 20, 21, 22]
VIT duration (years)	5	Aligned with SmPCs and EAACI guidelines	[6, 15, 16, 17, 18, 20, 21]
Clinical			
Utility decrement—HVDI			
Sting, with no systemic reaction	0.032	Fear is assumed to be alleviated by 60% when a person does not experience a systemic reaction after a sting due to VIT	Assumption
Sting, with a systemic reaction	0.08	Fear is assumed to be alleviated by 0% if a patient experiences a systemic reaction after having received VIT	Assumption
No sting	0.032	The fear linked to having a systemic reaction is assumed to be alleviated by 60% due to VIT. This was chosen as it was assumed that fear gradually will be reduced along the treatment process, this reasoning aligns with prior findings of a longer duration of VIT is associated with lower levels of anxiety [9, 23]	Assumption
Utility decrement—Adrenaline auto‐injector			
Sting, no reaction	0.08	This was based on the utility decrement associated with the anxiety of experiencing a systemic reaction in patients with food allergies. This decrement was assumed to be transferable to the utility decrement linked to the anxiety of an individual with Hymenoptera venom allergy experience	[24]
Sting, systemic reaction	0.08		[25]
No sting	0.08		[25]
Background utility—age‐adjusted	18–29: 0.871 30–39: 0.848 40–49: 0.834 50–69: 0.818 70–79: 0.813 80+: 0.721	The baseline value used for utility is based on the age‐adjusted utility weights of the Danish population (EuroQol‐5 Dimension). Mean age was set to 30 years	[24, 26, 27]
Transition probabilities			
Sting frequency	9.5%	The sting frequency during/following treatment with VIT frequency was based on a pooled average number from Hockenhull et al. [4]	[4]
Risk of systemic reactions	45%	The risk of having a systemic reaction depends on the time from the last reaction and the severity of the last reaction. To reflect the mean risk of systemic sting reaction in individuals with Hymenoptera venom allergy, 45% was chosen. This estimate was validated by clinical experts/coauthors	[19]
Protection against systemic reaction using HVDI	95%	Based on the available efficacy data from studies that exclusively employed HVDI with Alutard SQ in at least one treatment arm during both the up‐dosing and maintenance phases, and which followed up‐dosing protocols in accordance with the SmPCs	Aggregated, based on Appendix S7
Risk of systemic reaction with VIT	5%	From data described above, the efficacy of HVDI with Alutard SQ was found to be 95%, which leaves a risk of a systemic sting reaction after VIT of 5%	

Abbreviations: AAI, adrenaline auto‐injector; EAACI, European Academy of Allergy and Clinical Immunology; EMA, European Medicines Agency; HVDI, Hymenoptera venom depot immunotherapy; VIT, venom immunotherapy.

### 2.4 HVDI Efficacy

Several studies have investigated the efficacy of HVDI using Alutard^®^ SQ bee venom and/or wasp venom. The studies listed in Appendix S7 were chosen as data sources for the health economic analysis, as they provide efficacy data for HVDI and employed protocols similar to Danish clinical practice [20, 21]. The model does not distinguish between HVDI bee venom and wasp venom. Consequently, an aggregated efficacy estimate of 95% has been calculated, whereas the effectiveness is defined by the prevention of SSRs, with local reactions being deemed tolerable. This estimate is consistent with data from a larger population study with cross‐over from aqueous allergen solution to depot preparation and rush protocol, which found an efficacy of VIT at 98.5% [28].

### 2.5 Utility Decrements

To our knowledge, there is no published information on the utility decrement associated with the fear of experiencing a SSR. However, Voordouw et al. have estimated a 0.08 utility decrement associated with the risk of recurrence of anaphylaxis in subjects with food allergies based on EQ‐5D measured using the self‐reported visual analogue scale (VAS) format [25, 29]. This estimate has been chosen to represent the utility decrement linked to the risk of a SSR in individuals with bee and/or wasp venom allergy in the model. This utility decrement was assumed to be partially alleviated with VIT as previous studies have indicated that the level of anxiety decreases with the duration of VIT [9, 23]. The additional utility inputs have been based on assumptions. These utility inputs have been selected as they were deemed the most suitable alternative available, given the absence of specific utility decrements associated with SSRs. The decrements have been subtracted from an age‐adjusted baseline utility weight from the Danish population.

### 2.6 Costs

A Danish payer perspective has been utilized in the cost‐effectiveness analysis, encompassing healthcare sector expenses. In Denmark, immunotherapy is administered both in hospitals and specialized allergy clinics. The cost of the fees and expenses in a specialist setting has been retrieved from the Danish “intern medicin – takstkort 17A”. These tariffs^1^ are effective from April 1, 2025 and include the fee related to each injection against one allergen and an annual follow‐up consultation [30]. The pharmacy retail prices for HVDI using Alutard^®^ SQ in Denmark are as shown in Table 3, these were used for the medication expense of the total cost estimate.

**TABLE 3:** Costs related to treatment with HVDI using Alutard^®^ SQ bee and wasp venom, including the total cost.

Cost input	Cost	Use per injection
Vaccination against 1 allergen	€71.42	1
Annual follow‐up consultation	€81.76	N/A

Medication	Pharmacy retail price per pack	Doses per pack	Pharmacy retail price per dose
HVDI (bee venom)			
Up‐dosing phase	€525.58	1 up‐dosing scheme	€525.58^a^
Maintenance phase	€454.97	5	€90.99
HVDI (wasp venom)			
Up‐dosing phase	€530.18	1 up‐dosing scheme	€530.18^a^
Maintenance phase	€457.47	5	€91.49
Average cost			
Up‐dosing phase	€527.88	1 up‐dosing scheme	€527.88^a^
Maintenance phase	€456.22	5	€91.24

Treatment year	Total cost
Year 1	€2684
Year 1+	€1492
5‐year VIT treatment with HVDI	€8652

*Note:* Values were converted into Euro, medicine prices were obtained 13 January, 2025.

^a^ The cost of the up‐dosing pack was considered to be fixed regardless of the number of injections, so no price per dose is provided.

*Source:* References [30, 31].

The up‐dosing scheme consists of 15 weekly injections, followed by a maintenance phase with injections every 4–8 weeks, in alignment with the Danish summary of product characteristics [20, 21, 32, 33]. Assuming 6‐week intervals for the maintenance phase, the cumulative number of yearly injections during the maintenance phase is ∼6.2 injections for the first year, and ∼8.7 injections for the subsequent years. The cost of the administration fee was multiplied by the number of injections and added to the cost of the up‐dosing pack to find the cumulated cost of the up‐dosing phase. The cost of the maintenance phase was calculated by multiplying the number of injections by the cost per dose of the maintenance pack with the administration fee. The total costs, the costs of the up‐dosing year (year 1) and costs per maintenance years (year 1+) are presented in Table 3. In this analysis a 5‐year treatment duration with HVDI was chosen in alignment with the Danish treatment guidelines [14].

For the nonsystemic reactions, no additional costs are added, as it is assumed in this model these reactions do not require substantial treatment. To the systemic reactions, the cost of treating a systemic reaction in Denmark is added, which is estimated to be DKK 13,784.00 (€1847.67^1^) under the Danish diagnosis‐related group (DRG)–tariff system [34]. This cost estimate should be considered as an average, encapsulating various degrees of systemic reaction severity. The Pharmacy Retail Price of an EpiPen was €57.74 on January 13, 2025 [31]. The cost of one EpiPen is added to the AAI arm in the model each year to resemble having one AAI prescribed annually [35]. Additionally, the cost of one EpiPen is added to the cost in the up‐dosing phase in the VIT arm, as the Danish treatment guidelines recommend prescribing AAIs to patients undergoing the up‐dosing phase of VIT [14].

### 2.7 Sensitivity Analyses

Uncertainties of inputs in the model and their impact on the results were investigated using DSAs. The choices of inputs to vary were based on the inputs' inherent uncertainty as well as their variability within the relevant population in the model. The list of sensitivity analyses and the applied variances is listed in Table 4.

**TABLE 4:** Sensitivity analyses.

Parameter	Variation—upper and lower bound	Reason for choice of parameter and variation
Time horizon – duration of efficacy of VIT	10–15 years	A lower bound of 10 years, equivalent to the base case analysis, was selected based on the assumption that the duration of VIT is 5 years, with an additional efficacy period of at least 5 years after ended treatment. An upper bound of 15 years was explored under the premise that considering only 5 years of treatment efficacy is very conservative. Thus, it was deemed pertinent to assess the impact assuming a 10‐year efficacy period
Duration of VIT treatment	3–5 years	An upper bound of 5 years VIT equal to the base case was chosen, as a conservative approach, however a lower bound of 3 years VIT was investigated as the EAACI guidelines state 3 years treatment duration can be recommended, due to an evidence gap in the optimal duration [6]. In this scenario it was still assumed that VIT has 5 years efficacy, thus the time horizon was set to 8 years
Number of AAIs prescribed annually	2–4 AAIs	In the base case, one AAI is prescribed annually, to keep a conservative perspective. However, the impact of having two or four AAIs prescribed annually was investigated. This aligns with the EMA assessment that healthcare professionals preferably should prescribe two AAIs to each patient [36]
Utility decrements—Adrenaline injector: sting, with no systemic reaction	0.000–0.161 (101%)	These inputs were individually varied to identify the highest variation possible for each input before the ICER would cross a threshold. No cost‐effectiveness threshold is defined in Denmark, therefore the commonly cited cost effectiveness threshold of £20,000–£30,000 from NICE was utilized [37]. The lowest range of the threshold was used and converted from £ to € using the European Central Banks currency converter on May 20th, 2025, which equals €23,759. When an input could be varied with > 100% before crossing the threshold, it was chosen to vary this with 101%. This approach was employed as the utility inputs are associated with several uncertainties, mainly because they have been based on data from food allergy and assumptions regarding fear alleviation due to VIT was made
Utility decrements—Adrenaline injector: sting, with systemic reaction		
Utility decrements—Adrenaline injector: no sting	0.064–0.096 (20%)	
Utility decrements—VIT: sting, with no systemic reaction	0.000–0.064 (101%)	
Utility decrements—VIT: sting, with systemic reaction	0.000–0.161 (101%)	
Utility decrements—Utility decrements—VIT: no sting	0.014–0.050 (57%)	
Risk of systemic reactions in adults in the absence of VIT	10%–80%	This was varied from 10% to 80%, as the risk is highly dependent on several variables such as severity of last reaction and time in‐between stings, and therefore it is relevant to investigate the impact. This input was varied with a great range due to limited information on this estimate
Risk of systemic reactions with VIT	1.1%–8.9%, corresponding to a 91.1%–98.9% efficacy of VIT	Consistent with the risk of a systemic reaction in the absence of VIT, the variation in the risk of the VIT population was adjusted proportionally to the base case value

Abbreviations: EAACI, European Academy of Allergy and Clinical Immunology; VIT, venom immunotherapy.

## 3 Results

### 3.1 Base Case Analysis

Over a 10‐year period, the outcomes for HVDI compared to AAIs demonstrate that the cost in the HVDI arm is € 8796 with a QALY of 8.16, while the cost in the AAIs arm is €1367 with a QALY of 7.68.

The model estimates an incremental QALY of 0.48, corresponding to an additional 0.48 year in perfect health due to HVDI compared to AAIs. The incremental costs of HVDI are €7428. Consequently, the ICER (cost per QALY) of HVDI compared to AAIs after 10 years is €15,550. The results indicate that the initially higher costs associated with HVDI compared to AAIs are offset by significant health benefits by reducing fear associated with stings by decreasing SSR.

### 3.2 Sensitivity Analysis

#### 3.2.1 Deterministic Sensitivity Analysis

In Table 5, the chosen DSAs are listed, ranking them from the parameters with the largest impact on the ICER to the lowest. The sensitivity analyses resulted in ICERs between €9412 and €23,759. The three most impactful parameters in the DSA were the utility decrement for VIT—no sting, the utility decrement for adrenaline auto‐injector—no sting, and the duration of VIT, indicating that the base case result is robust but particularly sensitive to patient quality‐of‐life improvements, comparator anxiety levels, and the treatment duration reflecting Danish guidelines.

**TABLE 5:** Parameters' uncertainties impact on the ICER, sorted by highest impact.

Parameter	Deterministic	Lower bound	Higher bound	Base case	ICER using lower bound value	ICER using higher bound value
Utility decrement—VIT—no sting	0.032	0.014	0.050	€15,550	€11,556	€23,759
Utility decrement—adrenaline auto‐injector—no sting	0.080	0.064	0.096	€15,550	€23,725	€12,688
Duration of VIT	5 years	3 years	N/A	€15,550	€9305	N/A
Time horizon	10 years	N/A	15 years	€15,550	N/A	€9412
Risk of systemic sting reaction without VIT	0.450	0.100	0.800	€15,550	€17,871	€13,473
Utility decrement—adrenaline auto‐injector—sting, no reaction	0.080	0.000	0.161	€15,550	€17,057	€14,287
Utility decrement—adrenaline auto‐injector—sting, systemic reaction	0.080	0.000	0.161	€15,550	€16,761	€14,501
Number of AAIs	1	2	4	€15,550	€14,462	€12,286
Utility decrement—VIT –Sting, no reaction	0.032	0.000	0.064	€15,550	€14,655	€16,561
Risk of systemic reaction after VIT	0.050	0.011	0.089	€15,550	€15,316	€15,786
Utility decrement—VIT—sting, systemic reaction	0.080	0.000	0.161	€15,550	€15,426	€15,675

Abbreviations: ICER, Incremental Cost‐effectiveness Ratio; VIT, venom immunotherapy.

## 4 Discussion

In this study, we have estimated the ICER of VIT for the prevention of SSRs compared to AAIs. No cost‐effectiveness threshold is defined in Denmark but using the commonly cited cost‐effectiveness threshold of £20,000–£30,000 from NICE, VIT is cost‐effective against AAIs in the Danish treatment setting [37]. The sensitivity analyses related to the utility decrements were varied as much as possible while remaining below the NICE threshold, and none of the other sensitivity analyses exceeded the NICE threshold. Thus, the conclusion remained robust against the uncertainties concerning the inputs in all the sensitivity analyses, excluding the utility inputs as these were performed as threshold analyses. This indicates that costs related to VIT are offset by significant health benefits by reducing fear associated with stings by decreasing SSR.

Our findings should be considered in the context of the only prior cost‐effectiveness study of VIT by Hockenhull et al. [4], which assessed the now discontinued Pharmalgen from a UK payer perspective by modeling SSRs and sting‐related mortality into QALYs. The prior study reported very high costs per QALY gained for VIT compared with high‐dose antihistamines and AAIs, hence concluding that VIT was not cost‐effective except in very high‐risk populations such as beekeepers with frequent stings. Based on this evidence, the EAACI guidelines on allergen immunotherapy and a systematic review of health economic analyses of allergen immunotherapy have stated that VIT should mainly be considered in high‐risk groups with multiple exposures per year [6, 38].

In contrast, our analysis evaluates depot VIT with Alutard SQ under Danish conditions, incorporating both psychological and physiological benefits of treatment in QALYs, together with applying current Danish cost and practice patterns. These differences in intervention, outcome measures, and perspective may account for why our study indicates that VIT is cost‐effective compared with AAIs across all patients rather than only in very high‐risk groups. Hence, this comparison shows how our study complements and extends the existing evidence on health economic analyses of allergen immunotherapy, clarifying the circumstances under which VIT may be economically attractive, and highlights the relevance for future cost‐effectiveness analyses of VIT to incorporate psychological outcomes such as fear reduction alongside clinical outcomes. As the health‐economic model applied in this study is dynamic, it can be adapted to other European healthcare settings, thereby allowing the perspective to be extended beyond the Danish context.

Our study, however, has limitations because some of the inputs used in the health economic models are associated with significant uncertainties. Several sensitivity analyses were conducted to assess the potential impact of these uncertainties on the results. Furthermore, a conservative approach was adopted whenever feasible. One of the most important inputs is the efficacy duration of HVDI after completion of the maintenance treatment phase. No studies supply high‐quality evidence on the duration patients can remain off maintenance treatment while still benefiting from HVDI. The general consensus is at least 5 years of efficacy as adapted in the model. The result of the sensitivity analysis assuming an additional 5 years of efficacy resulted in a lower ICER, as the combined costs of AAIs near the total costs of HVDI over time without impacting the QALYs obtained in each arm. Consequently, in clinical practice, the longer patients can remain off maintenance treatment with HVDI but still benefit from the treatment, the more cost‐effective HVDI will be. When the treatment duration of VIT is shortened to 3 years from 5 years, HVDI becomes more cost‐effective. Likewise, when the number of AAIs prescribed is increased to 2–4 annually, the ICER also decreases in HVDI's favor.

The likelihood of experiencing a systemic reaction to a bee and/or wasp sting largely depends on the severity of the previous reaction and the duration between stings. As such, it was important to examine the effect of altering the risk of a SSR. Hence, the base risk of a SSR was varied from 45% to 10% and 80% after a sting in sensitivity analyses. Assuming a SSR risk of 10% meant an increase in the ICER of €2321 (14.9%). Conversely, assuming a SSR risk of 80% resulted in an ICER decrease of €2077 (13.4%), suggesting that the more at‐risk populations have a greater potential for cost‐effectiveness as would be expected.

For patients receiving VIT the risk of having a SSR was 5% due to 95% VIT effectiveness. Consistent with the risk of a SSR in the absence of VIT, the variation in the risk of the VIT population was adjusted proportionally to the base case value, which equals upper and lower bounds of a SSR after VIT at 1.11%–8.90%, respectively. Altering the probability of a SSR after VIT impacted the ICER insignificantly, as the risk remained small in both extremes. This highlights that the difference in efficacy between bee and wasp VIT would not impact the overall cost‐effectiveness of VIT [6].

The most significant limitation of our study was the lack of data sources for the utility of patients at risk of SSR. Consequently, we used a utility decrement associated with the fear of anaphylaxis in patients with food allergies as a proxy for the utility decrement associated with the fear of experiencing a SSR. Although this approach causes significant indirectness, compared to healthy individuals, both patient groups are significantly affected in terms of HRQoL [39]. The assumed utility decrement is substantiated by studies using the Vespid Allergy Quality of Life Questionnaire that have shown quality of life improved after VIT, and that a tolerated sting after having received VIT improves the quality of life [8, 40, 41, 42]. Supporting this, a longer duration of VIT is associated with lower levels of anxiety [9]. Altogether, these studies support the relative utilities for patients on VIT compared to AAI's in the context of this study.

Still, the exact utility decrement associated with being in fear of a systemic reaction without being stung remained uncertain. Therefore, sensitivity analyses on the utility inputs were conducted as threshold analyses to determine how much each input could be adjusted before the results exceeded the threshold. All utility inputs could be varied > 100% without changing the conclusion, except the inputs related to the population of patients who are not stung. Still, the “Utility decrement – VIT ‐ no sting*”* could be varied with 57% without exceeding the threshold, whereas the “Utility decrement ‐ adrenaline autoinjector ‐ no sting” could be varied with 20%. This illustrates the major difference between the two treatment strategies in that patients depending on AAIs live with considerable fear of a SSR, regardless of being stung, unlike patients receiving HVDI who rarely experience SSRs, potentially restoring at‐risk patients to a life less affected by anxiety and risk of SSRs [15, 16, 17, 18].

Another consideration related to the fear of a SSR is that bees and wasps are more active during the summer. However, this study assumed that the fear of a SSR remained consistent throughout the year. The rationale is that fear does not fluctuate logically based on seasons, as encounters with bees or wasps can occur outside the summer period or during travels.

In conclusion, HVDI is a cost‐effective treatment for the prevention of severe reactions to bee and/or wasp venom when compared to AAIs. Further studies of the impact of being at risk of a SSR on utility and more robust data on the long‐term effect of VIT are needed to strengthen the analysis presented in the study.

## Author Contributions

All authors contributed to data analysis and/or interpretation as well as to preparing and critically reviewing the manuscript. All authors reviewed the manuscript, revised the content, approved the final version for submission, and agree to be accountable for all aspects of the work. Ultimate responsibility for the opinions, conclusions, and data interpretation lies with the authors. Open Access funding enabled and organized by Projekt DEAL.

## Funding

The study was funded by ALK‐Abelló A/S (Hørsholm, Denmark).

## Conflicts of Interest

Dr. Sturm reports grants from ALK‐Abelló, personal fees from ALK‐Abelló, personal fees from Allergopharma, Novartis, HAL, and from Stallergenes‐Greer, outside the submitted work. Prof. Bilò received honoraria as a speaker from ALK‐Abelló, AstraZeneca, GlaxoSmithKline, Menarini, and Sanofi, outside the submitted work. Carmen Vidal has received funding for educational and research activities from ALK‐Abelló A/S, Allergy Therapeutics, AstraZeneca, GSK, HAL, Industry Roxal, Leti, and Stallergenes‐Greer. Joanna N. G. Oude Elberink is a member of the Advisory Board of Blue Print and PIMS Epinephrine. She has received consulting fees from Novartis, Behring, Viatris, Takeda, ALK‐Abello, Sanofi and Stallergenes. Prof. Schmitt reports institutional grants for investigator‐initiated research from the German Federal Joint Committee, German Ministry of Health, German Ministry of Research, European Union, German Federal State of Saxony, Novartis, Sanofi, ALK, and Pfizer. He participated in advisory board meetings as a paid consultant for Sanofi, Lilly, and ALK. Andreas Kallsoy Slættanes is an employee of ALK‐Abelló A/S. Prof. Dr. Thilo Jakob reports grants and personal fees from Allergy Therapeutics/Bencard Allergie, ALK‐Abelló, Galderma, HAL, Leo‐Pharma, Novartis, Sanofi‐Genzyme, and Thermo Fisher Scientific, outside the submitted work.

## Supporting information


**Appendices S1–S7:** Supporting Information.

## Data Availability

The data that support the findings of this study are available from the corresponding author upon reasonable request.

## Endnotes

^1^
Converted May 16th, 2025, using the European Central Banks currency converter; https://data.ecb.europa.eu/currency‐converter.
