# Supplementary material for: Cost‐Effectiveness of Venom Immunotherapy in Preventing Severe Bee and Wasp Sting Reactions
Source: Allergy. 2025 Dec 18;81(4):1228–38. doi: 10.1111/all.70176 (PMC13040657; doi:10.1111/all.70176)
Supplement: Supplementary file 1 — Appendices S1–S7: Supporting Information. [file ALL-81-1228-s001.docx]

|  | **PubMed** | **Embase** |
| --- | --- | --- |
| **Population** | (((((((((((("Venom Hypersensitivity"[Mesh]) OR "Wasp Venoms"[Mesh]) OR "Bee Venoms"[Mesh]) OR "Bees"[Mesh]) OR "Wasps"[Mesh])) OR (mastoparan)) OR (apamin)) OR (melittin)) OR ("bee sting")) OR ("wasp sting")) OR ("bee venom")) OR ("wasp venom") | 'hymenoptera venom allergy'/de OR 'wasp venom'/exp OR 'bee venom'/exp OR 'wasp'/de OR 'bee'/exp OR 'mastoparan'/de OR 'bee sting'/de OR 'wasp sting'/de OR 'venom hypersensitivity' OR 'wasp venoms' OR 'bee venoms' |
| **Outcome** | ((((((((((("Cost-Effectiveness Analysis"[Mesh]) OR "Health Care Costs"[Mesh]) OR "Costs and Cost Analysis"[Mesh]) OR "Resource Allocation/economics"[Mesh]) OR "Direct Service Costs"[Mesh]) OR "Hospital Costs"[Mesh]) OR "Health Expenditures"[Mesh]) OR ("budget impact")) OR ("immunization cost")) OR ("resource cost")) OR ("direct cost")) OR ("indirect cost") OR ("field sting") OR ("sting challenge") | 'cost effectiveness analysis'/exp OR 'health care cost'/exp OR 'resource allocation'/exp OR 'resource cost' OR 'immunization cost' OR 'budget impact' OR 'direct cost' OR 'indirect cost' OR 'field sting' OR 'sting challenge' |

Supplementary 1. Search strategy for costs search

Supplementary 2. Search strategy for resource use search

|  | **PubMed** | **Embase** |
| --- | --- | --- |
| **Population** | (((((((((((("Venom Hypersensitivity"[Mesh]) OR "Wasp Venoms"[Mesh]) OR "Bee Venoms"[Mesh]) OR "Bees"[Mesh]) OR "Wasps"[Mesh])) OR (mastoparan)) OR (apamin)) OR (melittin)) OR ("bee sting")) OR ("wasp sting")) OR ("bee venom")) OR ("wasp venom") | 'hymenoptera venom allergy'/de OR 'wasp venom'/exp OR 'bee venom'/exp OR 'wasp'/de OR 'bee'/exp OR 'mastoparan'/de OR 'bee sting'/de OR 'wasp sting'/de OR 'venom hypersensitivity' OR 'wasp venoms' OR 'bee venoms' |
| **Outcome** | (((((("Health Resources"[Mesh]) OR "Disease Management"[Mesh]) OR ("resource use")) OR ("hospital resource")) OR ("immunization resource")) OR (rush)) OR ("ultra-rush") | 'health care utilization'/exp OR 'disease management'/de OR 'resource use' OR 'hospital resource' OR 'immunization resource' OR 'rush' OR 'ultra-rush' |

Supplementary 3. PRISMA diagram of costs search


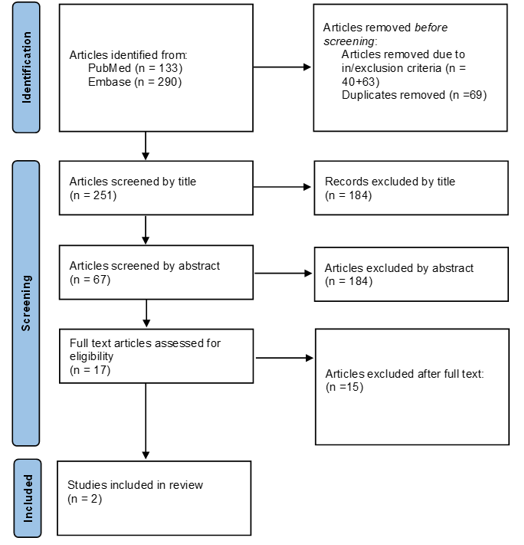


Supplementary 4. PRISMA diagram of resource use cost


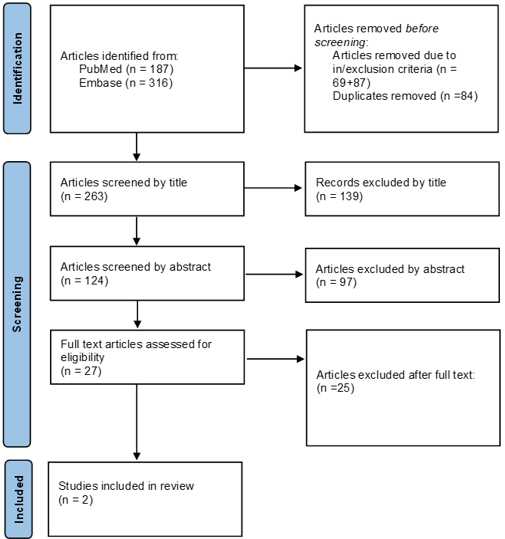


Supplementary 5 Overview of included studies from the ‘costs search’

| **Study** | **Inclusion reason** |
| --- | --- |
| **Title**: A systematic review of the clinical effectiveness and cost-effectiveness of Pharmalgen® for the treatment of bee and wasp venom allergy.  **Authors**: Hockenhull J, Elremeli M, Cherry MG, Mahon J, Lai M, Darroch J, Oyee J, Boland A, Dickson R, Dundar Y, Boyle R.  **Journal**: Health Technol Assess. 2012;16(12): III-IV, 1-110. | High quality study: systematic review.  Investigates the cost-effectiveness of Pharmalgen® thus, this provides relevant information to describe the cost related to bee and/or wasp allergy. |
| **Title:** Cost of testing and treating with Hymenoptera venom extracts.  **Author:** Lockey RF  **Journal:** J Allergy Clin Immunol. 1980 May;65(5):398-400 | The information is from a correspondence where Lockey, RF. writes to the editor and the information is from 1980. The text provides estimation of costs related to test and treatment of Hymenoptera sensitive individuals however, this estimation is described to be conservative. |

Supplementary 6. Overview of included studies from the ‘resource use search’

| **Study** | **Inclusion reason** |
| --- | --- |
| **Title**: Congruence of the current practices in Hymenoptera venom allergic patients in Poland with EAACI guidelines.  **Authors**: Cichocka-Jarosz E, Diwakar L, Brzyski P, Tobiasz-Adamczyk B, Lis G, Pietrzyk JJ  **Journal**: Arch Med Sci. 2011;7(5):832-9 | Study investigates congruence to EAACI guidelines in Poland. Provide information on diagnostic and therapeutic processes in Poland. |
| **Title**: Practice of venom immunotherapy in the United Kingdom: a national audit and review of literature  **Authors**: Diwakar L, Noorani S, Huissoon AP, Frew AJ, Krishna MT  **Journal**: Clin Exp Allergy. 2008;38(10):1651-8 | Study investigates practice of venom immunotherapy in UK. Provide information on current practice in diagnostics and treatment processes in the UK, which could be useful to estimate resource use related to immunotherapy. |

Supplementary 7. Overview of the efficacy of Alutard® SQ

|  | **Reactions to sting** | **Efficacy (%) in prevention of systemic sting reactions** | **Method of efficacy evaluation** |
| --- | --- | --- | --- |
|  | **Treatment efficacy for Wasp venom** | | |
| **Poli et al. (2001)**^41^ | Subjects only developed local mild erythema and no systemic reactions | 13/13 = 100% | Field sting |
| **Schrautzer et al. (2020)**^42^ | All subjects tolerated the sting | 73/73 = 100% | Sting challenge |
| **Alessandrini et al. (2006)**^43^ | They all experienced only local adverse effects consisting of limited local erythema that did not require any treatment. | 10/10 = 100% | Field sting |
|  | **Treatment efficacy for Bee venom** | | |
| **Rueff et al. (2004)**^44^ | No systemic reactions were developed to field stings. One subject developed grade I reaction and two developed grade II according to Ring and Messmer classification following the sting challenge | 15/18 = 83.3% | Combined: 4 field stings and 14 sting challenges |
| **Quercia et al. (2006)**^45^ | Subjects only developed local discomfort at the site of the sting and no systemic reactions | 15/15 = 100% | Field sting |
|  | **Treatment efficacy for Bee and Wasp venom reported combined** | | |
| **Wyss et al. (1993)**^46^ | One subject treated for bee venom developed mild allergic symptoms of grade II (only after a third sting) according to Mueller’s classification | 10/11= 90.9% | Field sting |
| **Çetinkaya et al. (2018)**^47^ | 19 subjects developed local, one developed a large local and 4 developed systemic reactions (grade I or II according to Mueller's classification) | 29/33=87.9% | Field sting |
| **Cadario et al. (2004)**^48^ | The subject developed a mild local reaction which did not require any treatment. | 6/6 = 100% | Field sting |
| This overview does not differentiate between the type of up-dosing protocol.  * The reported efficacy in participants receiving either Alutard® SQ (n= 103) or Alyostal® (n= 4) were not differentiated. | | |  |
